# Meetings of Societies

**Published:** 1901-06

**Authors:** 


### MEETINGS OF SOCIETIES.

### Bristol Medico-Chirurgical Society.

*March 13th, 1901.*

Dr. D. S. Davies, President, in the Chair.

Dr. Michell Clarke showed the following pathological specimens: (1) Sections of the brain obtained from a case of tumour of the right optic thalamus and crus cerebri. The sections showed the extent and relations of the tumour. (2) Specimens obtained from a case of tumour of the right cerebral hemisphere involving the corpus callosum. The patient, a woman aged 31, had been shown to the Society some months previously. The tumour was a round-celled sarcoma. The symptoms had begun with Jacksonian epilepsy affecting the left side. There was no optic neuritis at that time. Twelve months later there was left hemi-paresis and slight optic neuritis. Operation was then declined. Three or four years from the onset of the disease she came to the Hospital again with more marked hemiplegia and almost complete loss of sight. It was decided to perform an exploratory operation. The patient unfortunately died from the shock and hemorrhage which attended the operation. (3) A specimen beautifully illustrating the lesion in that form of endemic dysentery or ulcerative colitis met with in this country.—Dr. Lacy Firth, referring to the second case mentioned above, stated that he had carried out the operation alluded to. A quadrilateral flap had been raised and a large piece of bone removed. Before operating a tourniquet had been applied round the head with the object of diminishing hemorrhage, but his impression was that the tourniquet had rather increased the hemorrhage than diminished it, in spite of the fact that it had been retightened after the first incision. The patient bore the operation well until near its completion, when her condition suddenly became critical. She lived about eight hours after the operation. Just before death she was transfused. During the transfusion she had a generalised epileptiform fit, and then stopped breathing from paralysis of the respiratory centre. The dura mater was incised as a last resort to relieve intracranial tension, but without benefit.

Dr. T. Fisher, whose paper we shall print in full in a later issue, showed a series of sketches and diagrams of displaced abdominal organs. They illustrated the following conditions: (1) Traumatic diaphragmatic hernia, taken from the case of a man who had been squeezed between the foot-board of a train and a station platform. The stomach and part of the transverse colon had entered the left side of the thoracic cavity through the œsophageal and aortic openings, which had been ruptured into one aperture. (2) Congenital diaphragmatic hernia in a child seven months old. The usual attachment of the external arched ligament was absent on the left side, and through the gap all the intestines between the third part of the duodenum and the descending colon had passed into the left side of the thoracic cavity. The stomach had remained in the abdomen. (3) The cæcum attached to the lumbar vertebræ below the duodenum, from a man aged 38. The appendix, directed upwards and to the right, reached the costal margin. (4) A cæcum with a long meso-cæcum lying on the anterior surface of a slightly enlarged liver, from a woman aged 35. The cæcum could be placed in the left iliac fossa. (5) The transverse colon and part of the stomach turned upwards and lying on the anterior surface of the liver, from a woman, aged 54, who died of carcinoma of the cæcum. (6) A transverse colon adherent to the back of the uterus, with the small intestine in front, from a case of subacute peritonitis in a woman of 29. (7) Acute dilatation of the stomach in a case of septicæmia, from a woman aged 36. The stomach reached the pubes and left iliac fossa. (9) A greatly dilated transverse colon, from a case of carcinoma of the sigmoid flexure. On first opening the abdomen nothing but this and the stomach adherent to it could be seen. (10) A diagram of Riedel's lobe of the liver taken from a man aged 46. This sketch had not been made from a *post-mortem* subject, though the speaker had twice met with the abnormality in the *post-mortem* room. Though this was not an illustration of a displaced viscus, it was a condition which had been frequently mistaken for movable kidney.—Dr. James Swain commented upon the importance of some of the conditions which had been illustrated. Riedel's lobe was most apt to be mistaken for a distended gall-bladder.—Mr. C. A. Morton commented upon the V-shaped colon and the adhesions which had caused it.—Dr. Michell Clarke remarked that he had often seen a V-shaped colon, and asked what had been the physical signs in the case of congenital diaphragmatic hernia.—Mr. Carwardine stated that he had seen a laparotomy performed for what proved to be Riedel's lobe of the liver. He had seen the lobe also in a liver he had operated upon for a hydatid cyst. He alluded also to a case in which the cæcum had been found on the left side of the abdomen.

Mr. C. A. Morton read notes of a case of successful operation for diffuse peritonitis from perforation of the appendix vermiformis. He remarked that cases had been described as examples of general peritonitis in which there had been no evidence that inflammation was general throughout the peritoneum. His own case was an example of diffused peritonitis, and probably the inflammation had not spread to the diaphragm. The case appeared to teach that cases of appendicitis, especially if recurrent often, could not be safely left for operation until a quiescent interval was reached. The patient, a man of forty-four years of age, awoke one morning with some abdominal pain. This became severe on a railway journey to Bristol. Mr. Morton had seen him a few hours later, and found him with a pulse of 80, a temperature of 100°, rigidity of the right side of the abdomen, and tenderness at a point over the right iliac fossa to the inner side of the iliac spine. This was on December 4th. There was a history of three previous similar attacks, the last one having been in October. It was thought the case might be safely watched. On the next day the pain had spread to the left of the abdomen, and there was slight abdominal distension, but no vomiting. Forty-eight hours after the onset there was more general tenderness, and the abdominal movements of respiration had stopped; operation was then decided upon. Pus was found beneath the cæcum, apparently confined there only by the pressure of distended coils of intestine. A large quantity of thick pus was found in the pelvis. The appendix was almost free from adhesions, and was removed. The exposed intestine was very red, and had flakes of lymph adherent to it. Three drainage tubes were inserted in different directions. These were removed in five days. Ten days after the operation the patient was able to take solid food. The sinus left gradually closed. Commenting on this case, Mr. Morton said that some members of the profession did not realise how dangerous recurrent cases of appendicitis often were. In such cases, even with the pulse only slightly accelerated, with little distension, and with no vomiting, it might be dangerous to put off operation.—Distension of the abdomen does not prove that a peritonitis is general. Dr. James Swain remarked that the term "spreading peritonitis" was a suitable one for those cases of peritonitis starting from a definite focus like the appendix, and that a true general peritonitis rarely resulted. During the period when the inflammation was spreading an operation might cure the patient. If the irrigation treatment was used a spreading peritonitis might be generalised. He considered that in appendicitis each case should be treated on its own merits, and he would wait for the quiescent period before operating in any case in which it appeared likely that the patient would get over the acute attack.—Dr. Watson Williams alluded to the difficulty there was in deciding whether a case was one of diffuse or general peritonitis, and whether, in the severest cases, an operation afforded hope or was hopeless.—Mr. Paul Bush agreed that the "flushing-out" treatment of peritonitis often spread the disease, and believed that patients had been killed by it. He advocated thorough sponging and free drainage. —Mr. Lace asked if Mr. Bush was averse to irrigation in general peritonitis. He had been in the habit, if he found pus around the appendix and in the pelvis, of making an incision above the umbilicus, and, if he found peritonitis there, of irrigating.—Mr. Carwardine pointed out that general peritonitis involved the under-surface of the diaphragm, and the region behind the stomach, and the lesser sac of the peritoneum, and that the term should not be used unless these parts were involved. He alluded to the method of turning out the intestines and thoroughly cleansing them in that way.—Mr. W. H. Harsant said he would be sorry to lay down the rule that the method of irrigation should never be used. A general rule against the practice should not be made, but each case judged on its merits. It was certain that flushing was now less practised than formerly.—Dr. W. C. Swayne mentioned a case in which a woman had died six months after confinement from rupture of an abscess from the peritoneum into the lung through the diaphragm, and in which a score of pockets of pus was found amongst the intestines. He would be disposed to irrigate in such a case, and had, in fact, done so in two cases, though unsuccessfully. Irrigation was the only possible way of dealing with such cases. In some the whole peritoneal cavity became affected in twenty-four hours.—Mr. Morton referred to the presence of rectal tenesmus in some cases. In the very severe cases operation was almost hopeless. He agreed with Mr. Lace upon the advisability of making a second incision at a distance from the appendix region. Some remarkable recoveries had followed the treatment by turning out the intestine and then cleansing the peritoneum.

Dr. Bertram Rogers read notes on a case of acute pemphigus in which Demme's diplococcus had been found by Dr. Neild. The patient was a child. The illness had begun with a sore throat. The tonsils and uvula had a sloughy appearance. Diphtheria antitoxin had been used. Numerous blisters—thirty or forty—formed on different parts of the body. The child died with broncho-pneumonia. —Dr. Symes remarked that he had seen the case at the Hospital, and regarded it as one of septicæmia. He did not attach much importance to the organism obtained from surface blebs, and considered the examination of the blood more important. The eosinophile cells had been almost as numerous as the leucocytes. The question as to whether pemphigus was really a specific disease or not was alluded to.

---

*April 10th, 1901.*

Dr. D. S. Davies, President, in the Chair.

Mr. T. Carwardine showed a patient after operation for a large ventral hernia. It was explained that the patient had in the first instance been successfully operated upon for a ruptured liver, and that a ventral hernia had formed through the abdominal cicatrix. The operation for the relief of this condition had consisted in exposing the affected parts and suturing each of the layers of the abdominal wall accurately together where they had gaped. In this way the posterior layer of the rectus sheath, the rectus muscle itself, the anterior layer of its sheath, and the skin had been successively sutured. About fifty buried and twenty superficial sutures had been used, and the operation took about an hour and twenty minutes. Lembert's sutures had been used for the anterior layer of the rectus sheath. The result now, many months after the operation, was a strong abdominal wall. A slight degree of atrophy of the upper part of the rectus muscle remained.—Dr. J. Lacy Firth said that he remembered well seeing the large ventral hernia the patient had when he appeared before the Society after his recovery from the first operation. He had now examined him again, and considered the result obtained by the operation for the ventral hernia was a most excellent one. He asked if at the operation the old incisions had been opened up to their full extent, both the longitudinal one and the transverse, whether peritoneal adhesions had given any difficulty, what material had been used for the buried sutures, what material the operator generally preferred in operations for the cure of herniæ, and whether the opportunity had been taken to examine the scar in the liver, the result of its rupture. The rupture, as far as he remembered, had nearly completely separated the left lobe of the liver.—Mr. T. Carwardine replied that the old wounds had been completely re-opened; that the operation had practically and intentionally been an extra-peritoneal one, so that the liver could not be examined. Catgut had been used for the buried sutures, though, as a rule, he preferred silk for hernia cases. He was unable to see the advantage of using silver wire, as advocated by some.

Dr. Walter C. Swayne read a paper on a series of cases of cœliotomy for diseases of the uterus and ovaries. The series included sixteen abdominal sections and five vaginal sections. The former group included one exploratory operation, one for puerperal peritonitis, five ovariotomies, four oöphorectomies, one salpingo-oöphorectomy, and four hysterectomies with retro-peritoneal treatment of the stump. The vaginal operations included three hysterectomies for malignant disease, one vaginal section for general peritonitis, and one vaginal fixation for retroversion. Five of the patients had died, including the cases of puerperal peritonitis. Two cases of abdominal hysterectomy died—one from hemorrhage, the other from intestinal obstruction. One vaginal hysterectomy died of pneumonia. During the last twelve months the Trendelenbürg position had been used for all cases, and been found very advantageous. When the operation was a long one or attended with shock, as much boiled saline fluid as it would hold was poured into the abdominal cavity before tying the last stitch. In ovariotomy the proximal part of the ovarian artery was ligatured in the infundibulo-pelvic fold, and usually the cut ends of the artery were tied as well. Other details of technique were described. An account was then given of the various cases. One of the cases of hysterectomy vomited persistently for three days, and the patient's condition gave rise to some anxiety. All the unfavourable symptoms disappeared after washing out the stomach. The question as to the advisability of operating very soon after first seeing patients who were greatly weakened by malignant disease, or of deferring the operation until the patient's condition had been improved, was mentioned. There was more likelihood of dangerous shock and of sepsis and pneumonia if the operation was done immediately, and more likelihood of recurrence if it was delayed. The dilemma was most difficult. Allusion was made to the difficulty of keeping patients in the Trendelenburg position well clothed, so as to avoid shock. The value of strychnine injections, and of saline solution used as indicated above, were emphasised.—Mr. Ewens remarked upon the great recuperative power some patients showed after laparotomy in bad cases, and instanced a case in which he had evacuated at least a gallon of pus from the abdominal cavity with a successful result, though the operation was performed in a cottage.—Mr. Paul Bush remarked on the two cases in which the abdomen had to be re-opened for hemorrhage. He had seen the washing out of the stomach act like magic in checking vomiting.—Dr. Stack asked whether amenorrhœa had followed in one of the cases mentioned.—Dr. Lacy Firth was also interested in the case Dr. Stack had mentioned. It was the case in which a small portion only of one ovary had been left at the operation. The use of the word "peritonism" for a set of symptoms other than those of peritoneal shock, which Dr. Swayne had mentioned, seemed objectionable. Surgeons were often met with dilemmas such as that which had been alluded to in discussing the question of operating for malignant disease, and much judgment had to be used in deciding how to act.—Dr. Walter Swayne replied that he believed the patient inquired about had continued to menstruate after the operation. "Chronic peritonism" was the term he had alluded to as being occasionally used to signify the condition of abdominal pain and distension, the result of peritoneal adhesions.

Mr. Munro Smith read a paper written by Mr. A. W. Prichard, who was himself unavoidably absent, on some "operations undertaken for the closing of an artificial anus" (*see* p. 119).—Mr. T. Carwardine commented upon the excellent result of the treatment. There was now a great tendency to resect the gangrenous gut in these cases, and to immediately suture the divided parts. Statistics showed the results of this practice were much more favourable now than they used to be. A ten per cent. superiority in results was claimed by Americans when the Murphy button was used instead of sutures for joining the intestines, no doubt a result of the superior mechanical ingenuity of the American race.

Dr. Stack read notes of a case simulating plague. The patient, a man of twenty-three, was admitted to the Bristol Royal Infirmary under Dr. Shaw. The patient had previously attended as an out-patient for some weeks, suffering from plumbism. On January the 24th his face became very red and swollen, and he was delirious. Next day the swelling had spread to his neck, his mouth became very foul, and a red rash appeared on most of the body. The rash disappeared in about twenty-four hours. The patient then looked like a severe case of enteric fever in the third week. The temperature was 103°, respiration 32, and the pulse 100. There were signs of consolidation at the base of the right lung. The face and neck then peeled, and looked as if the patient had just had a severe attack of erysipelas. There were enlarged glands in the neck. On the sixth day after the face had first become swollen there was enlargement of the cervical, occipital, axillary, epitrochlear, and inguinal lymphatic glands, and they were tender. The man died eight days later. The temperature had remained high to the end, and there had been a gradual increase of prostration. Widal's reaction was negative. The blood and fluid obtained from the lymphatic glands produced no growth on agar tubes. A cover-slip preparation of a scraping from a lymphatic gland after death showed a short fat bacillus, which looked like a diplococcus owing to marked bi-polar staining. The case seemed to have been one of toxæmia, but the nature of the poison remained a clinical mystery.—The President said the case was one of considerable interest from a public health point of view. On January 20th a ship had come to Bristol upon which there were found rats which had died from plague. Dr. Davies had been interested, therefore, in discovering whether the man could have had anything to do with this ship, but he had found nothing pointing to that. The absence of signs of peri-glandular swelling was unlike the classical description of a plague case. In the single plague case which occurred at Cardiff there had been an absence of this peri-glandular swelling. He had formed the opinion that the man was not suffering from plague. The final criterion must be the bacterial one. Unfortunately the bacterial test was not a simple one, just as it was not a simple one in the case of diphtheria or tubercle. There were organisms which might be confused with that of plague.—Prof. Stanley Kent remarked that his introduction to the case was through an examination he had made of smears from the glands. In these he had found a stumpy bacillus which showed bi-polar staining. Cultures obtained from plague cases usually were pure, and showed the presence of the organisms in enormous numbers. He had failed to get any cultures from lymphatic gland substance in this case, but had obtained some from the spleen which were of a mixed character. In these cultures some of the organisms were like those of plague. Points against the bacillus being that of plague were the paucity in number of the organisms got from the lymphatic glands and the impossibility of making cultures of them. In favour of plague were the facts that the organism was contained in the glands, and that a pure culture had been obtainable from the spleen. An inoculated guinea-pig had failed to die. But occasionally in plague the poison was so attenuated that a large dose was required to kill a guinea-pig. Prof. Kent exhibited under the microscope specimens of the culture obtained from the spleen in the case of the typical bacillus pestis, and Dr. Stack's stained smears from the lymphatic glands.—Dr. Fletcher, who had seen the plague cases recently in Glasgow, said that the worst cases there had not been diagnosed at first, but had been notified as typhoid cases with a big query. It was the doubtfulness of the diagnosis which had led the hospital doctors to specially examine them. They concluded they were plague cases. The onset of the disease was not characteristic. Most of the temperature charts showed a well-marked primary and secondary fever; the late fever was coincident with sloughing of the glands. In an average case the glands gave great pain, and there was much hardness and redness over them. The character of the growth in agar was against the case described having been one of plague. Gentian violet had been the stain found to act best in Glasgow. A bacillus was always well demonstrable. In agar to agar culture the bacillus became less in size. There had been ten deaths in eighteen cases. The collapsed appearance and the look as of impending death which the patients exhibited had been a striking feature in the cases. In some of the most severe cases the temperature had not been very high.—Dr. Fisher alluded to the suggestion that there had been some very mild cases of plague about which were easy to overlook.—Dr. Stack reiterated that the man's appearance at first was that of a patient who had had erysipelas. With regard to the rash, they had only the description of the man's wife to guide them.

---

*May 8th, 1901.*

Dr. D. S. Davies, President, in the Chair.

Dr. J. O. Symes showed specimens of blood containing malarial organisms. They had been taken from a sailor who had his first attack of ague at Glasgow, eighteen days after leaving Egypt and eight days after leaving the South of Spain. At the beginning he had attacks of ague every day for ten days. Then they ceased for ten days, but after that returned daily. The films showed two generations of tertian parasites, and a marked decrease of the white cells. When the blood was again examined four days after the last attack of ague it showed an increase of the white cells and the presence of myelocytes. The count was as follows :—

| | | |
|---|---|---|
| Finely granular leucocytes ... ... | 52 | per cent. |
| Eosinophile leucocytes... ... ... | 3.6 | ,, |
| Myelocytes ... ... ... ... | 8 | ,, |
| Eosinophyle myelocytes ... ... | .3 | ,, |
| Large hyaline cells ... ... ... | 17 | ,, |
| Small lymphocytes ... ... ... | 19 | ,, |

The patient had been given grs. x. of quinine four times a day. The final attack of ague occurred after the second dose of quinine.

Dr. Aust Lawrence read a paper on "Some of the Hemorrhages of Pregnancy." (This paper we hope to publish in our next issue.)

Dr. Barclay Baron reported a case of paralysis of the right vocal cord in which the paralysis was the result of the pressure of a thoracic aneurysm, and showed skiagrams of the case. The patient, a man of fifty, had a piping voice and immobility of the right vocal cord on phonation. Also the left pupil was dilated and the left radial pulse was smaller than the right. There was a slight fulness above the right clavicle and the veins were rather enlarged there. Impaired percussion resonance was found over the upper three intercostal spaces on the right side, but no pulsation or bruit was detected. Over a limited area posteriorly, opposite the centre of the dull area in front, tubular breath sounds were heard. The fluorescent screen image and skiagrams, produced by Mr. James Taylor, showed the aorta projecting to the left of the spinal column, and a large dark shadow to the right; and on the screen distinct pulsation of these parts had been seen. The case taught the lesson that no patients should be allowed to go about with interference of function of the vocal organ without a laryngoscopic examination being made; otherwise such causes of the trouble as instanced above, and early stages of syphilitic or tubercular disease, or tumours, were likely to be overlooked.—Dr. Markham Skerritt remarked upon the successful use of skiagraphy in the case, and upon the difficulty there was in determining which blood-vessels or which parts of the vessels were primarily the seat of the disease in cases of aneurysm in the upper part of the thorax.—Dr. Watson Williams thought this was one of the first, if not the first case in which the diagnosis of aneurysm, suggested by laryngoscopy, had been confirmed by skiagraphy. Laryngologists had frequently diagnosed aneurysm correctly, but confirmation had in the past to be waited for until other symptoms developed. He agreed as to the necessity of making a laryngoscopic examination in any case of hoarseness or vocal change.—Mr. W. H. Harsant asked if the usual signs of aneurysm had been absent, so that diagnosis was impossible without a skiagram.—Mr. Paul Bush remarked on the perfection of the Röntgen ray demonstration of the nature of the case.—Dr. F. H. Edgeworth alluded to another case which he had seen in which abductor paralysis had been discovered in a patient attending the Bristol Royal Infirmary for eczema, and a skiagram had demonstrated the presence of a sacculated aneurysm.—Dr. K. Wills asked if there had been any dysphagia.—Dr. Baron replied that there had been no dysphagia and no pulsation, that the patient's voice was not hoarse but of a weak, high-pitched and "piping" character. The fluorescent screen had shown the aneurysm wonderfully well, even better than the skiagrams which had been handed round.

Dr. J. Michell Clarke read a paper on "The Treatment of Inveterate Cases of Sciatica." The treatment he described was that advocated and perfected by Weir Mitchell. It consisted in immobilization of the affected limb by means of bandages and a Liston's long splint. The limb was first bandaged in flannel from the ankle to the groin, and then the splint applied. For three or four days the splint was left undisturbed. After that it was reapplied daily, to make it more comfortable for the patient. After six or seven days slight movements of the hip and knee were made daily when the splint was removed. The duration of this treatment with the splint depended upon the severity of the case, but was usually two or three weeks. It was left off first by day for a week, and then altogether. After another week the flannel bandage was left off. A few days later the patient was allowed to get up, but not to sit down at all when up. The bandage was left off at first by night, and then altogether. If pain returned on getting up, part of the treatment had to be repeated. The patients felt the treatment very irksome for the first two or three days, and might even require morphia. Careful attention to details was essential for success. Arsenic, quinine, iron, or cod-liver oil might be used as adjuvants. The average duration of the treatment was six or seven weeks. The speaker had been successful in curing ten cases of very severe or obstinate sciatica by this method, and had found it more successful than any other treatment for that class of case. He recommended blistering any remaining tender areas.—Mr. H. Mole remarked that if the success of the splint treatment depended upon rest, a plaster-of-Paris splint might act as well as the Liston, and the patient be allowed up throughout.—Mr. W. H. Harsant considered perfect rest was essential for the cure of acute sciatica. The treatment described was in its essence that by perfect rest. It was an application of Hilton's principle of rest to cure pain.—Dr. Edgeworth was not surprised to hear that the patients found this method of treatment irksome. A peculiar feature in sciatica was that the patient could not keep the leg in one position. It had been alleged that the cremasteric reflex was increased on the affected side, which pointed to the existence of irritation of the lumbar region of the cord. In acute cases he applied a big blister, and gave salicylates. He thought the blister was useful, and was positive that the effect of salicylates was powerful and beneficial in severe cases. In chronic cases, such as those met with in hospital out-patient practice, he believed that giving one-hundredth of a grain of phosphorus three times a day did more good than anything. He had found it cure a case in a week which had previously been very persistent. Application of the constant current was also beneficial after the method of Lewis Jones.—Mr. Flemming mentioned a case he had relieved by using a Liston's splint for ten days. He had not found the constant current of much use. He had met a practitioner who advocated blistering the foot. Possibly that acted by inducing rest.—Dr. Elliott relied considerably upon salicylates and antipyrin. He thought the actual cautery also was useful.—Dr. Baron alluded to a case of frequently-recurring sciatica which a great many different drugs had failed to cure, but in which the Turkish bath, taken regularly, and the hot douche had given permanent relief.—Dr. Parker commented upon the treatment by daily painting the skin with concentrated hydrochloric acid over an area six or eight inches square. It rarely caused any injury to the skin.—Mr. Hancocke Wathen alluded to the use of an ether spray and of tetrachlorate of carbon.—Dr. Michell Clarke thought salophen a better drug for sciatica than salicylates. He did not think that a plaster-of-Paris splint would secure sufficiently perfect immoblisation if the patient was allowed up.

Dr. George Parker read a paper on a case of "Congenital Obliteration of the Bile Ducts," and showed the specimen obtained from the case. The patient was a male child six months old. The mother was healthy. She gave no history of syphilis. She had suffered from dyspepsia during pregnancy. The meconium at birth was natural, and the stools were natural until the third week of life. Then vomiting began, and a few days later jaundice with white stools was noticed. At this time the infant appeared bright and lively, the jaundice apparently being the only ailment present. Later the abdomen became slightly distended. There was some skin irritation about the arms. It was admitted to the hospital. Death occurred suddenly six months after birth. A little ascites had been detected. The stools contained a little blood for a day or two, and they changed from white to a yellow colour in the course of the disease. The urine contained bile, albumen, and bile-stained casts. At the *post mortem* the liver was found to weigh 11½ oz., double the weight for a child of that age. It was olive-green in colour. The section-surface was smooth. There was a thickening of the fibrous tissue in the hilum. No calculi were present. On cutting into a dilated duct clear fluid was found and a black deposit on the duct wall. The spleen was enlarged, dark, and firm, and weighed 5 oz. The common bile duct could not be traced to the pancreas. There was a considerable mass of connective tissue around the vena porta. The contributions of Rolleston and Thompson to the literature of the subject were alluded to. About sixty cases had been recorded. Three had been operated upon. Treves had operated on one patient nineteen years of age with this affection. The infants usually died before they were eight months old. There were three theories as to the origin of the affection, viz.:—(1) That it was due to malformation (obliteration) of the bile ducts; (2) That a toxin set up cholangitis, which was followed by obliteration of the ducts; (3) That cirrhosis began in the liver and obliteration of the ducts followed. The first theory is disproved, because patent ducts had been found in some cases. Rolleston suggested that a toxin passed to the fœtus along the umbilical vein, setting up multilocular cirrhosis. Biliary cirrhosis followed, due to the poison passing to the liver through the hepatic artery and being excreted by the ducts. Why should obliteration of the ducts ensue in children and not in adults? Possibly because of the small size of the ducts in the former. Similarly laryngitis might more often produce stenosis in children than in adults. There was a good deal of evidence that the liver disease was prior to the duct disease. No definite change had been found in the bile. The disease had not been traced to alcohol.

J. Lacy Firth.

